# Built Environment and Physical Functioning in Hispanic Elders: The Role of “Eyes on the Street”

**DOI:** 10.1289/ehp.11160

**Published:** 2008-05-28

**Authors:** Scott C. Brown, Craig A. Mason, Tatiana Perrino, Joanna L. Lombard, Frank Martinez, Elizabeth Plater-Zyberk, Arnold R. Spokane, José Szapocznik

**Affiliations:** 1 University of Miami Miller School of Medicine, Miami, Florida, USA; 2 University of Maine College of Education and Human Development/University Center for Excellence in Developmental Disabilities, Orono, Maine, USA; 3 University of Miami School of Architecture, Coral Gables, Florida, USA; 4 Lehigh University College of Education, Bethlehem, Pennsylvania, USA

**Keywords:** aging, built environment, environmental measures, Hispanics/Latinos, physical functioning, psychological distress, social support

## Abstract

**Background:**

Research on neighborhood effects increasingly includes the influences of the built environment on health and social well-being.

**Objectives:**

In this population-based study in a low-socioeconomic-status (SES), Hispanic neighborhood, we examined whether architectural features of the built environment theorized to promote direct observations and interactions (e.g., porches, stoops) predicted Hispanic elders’ social support and psychological and physical functioning.

**Methods:**

We coded built-environment features for all 3,857 lots in the 403-block area of an urban Miami, Florida, community. We then conducted three annual assessments of social support, psychological distress, and physical functioning in a population-based sample of 273 low-SES Hispanic elders (70–100 years of age). We used structural equation modeling analytic techniques to examine hypothesized relationships between the built environment and elders’ social support, psychological distress, and physical functioning over a 3-year period.

**Results:**

After controlling for age, sex, and income, architectural features of the built environment theorized to facilitate visual and social contact had a significant direct relationship with elders’ physical functioning as measured 3 years later, and an indirect relationship through social support and psychological distress. Further binomial regression analyses suggested that elders living on blocks marked by low levels of positive front entrance features were 2.7 times as likely to have subsequent poor levels of physical functioning, compared with elders living on blocks with a greater number of positive front entrance features [*b* = 0.99; χ^2^ (1 df) = 3.71; *p* = 0.05; 95% confidence interval, 1.0–7.3].

**Conclusions:**

Architectural features that facilitate visual and social contacts may be a protective factor for elders’ physical functioning.

A growing body of evidence suggests that the physical characteristics of neighborhoods are inextricably linked to residents’ behavioral and health outcomes [e.g., physical activity (Frank et al. 2004; Institute of Medicine 2005), cardiovascular disease (Diez Roux 2003; Ewing et al. 2003), and depression (Berke et al. 2007; Evans 2003)]. Aspects of the built environment such as mixed land use, moderate density, and connectivity have been shown to be related to greater “walkability” at the neighborhood level (Frank et al. 2004; Institute of Medicine 2005), enhanced community social processes (Leyden 2003), and more positive physical health outcomes (Diez Roux 2003; Ewing et al. 2003; Frank et al. 2004) and mental health outcomes (Berke et al. 2007; Szapocznik et al. 2006).

We examined a further aspect of the built environment theorized to influence health (Spokane et al. 2007) and social processes (Jacobs 1961): “eyes on the street” (Jacobs 1961), defined as architectural and neighborhood design features that promote direct observation and interaction among individuals in a neighborhood (Jacobs 1961; Leccese and McCormick 2000). Architectural features (e.g., porches, stoops, windows, and buildings sitting along sidewalks just above street level) are theorized to facilitate social interactions and monitoring of behavior among residents, which in turn promote social capital (Frumkin et al. 2004; Leyden 2003), social support, and social responsibility (Jacobs 1961; Leccese and McCormick 2000; Leyden 2003). Such outcomes are desirable, given the strong evidence that social support and positive neighborhood social environments are associated with a variety of health and mental health outcomes (House et al. 1988; Kawachi 1999; Krause et al. 1989; Sampson et al. 1997; Spokane et al. 2007).

The relationship between the built environment and health may be particularly important for older adults, who conduct most of their daily activities in their local environment (Horgas et al. 1998; Krause 1998). However, few studies have examined the neighborhood built environment in relation to elders’ health outcomes (Balfour and Kaplan 2002; Clark and George 2005).

## Built environment and physical functioning

Emerging evidence suggests that the neighborhood built environment may affect elderly residents’ physical functioning, defined as physical capabilities such as strength and mobility that are necessary for performing daily activities. For example, elders’ independence in activities of daily living was shown to vary as a function of built-environment attributes such as housing density and land-use diversity (Clarke and George 2005), features that may provide or restrict possibilities for pedestrian travel and related social interaction (Berrigan and Troiano 2002; Frank et al. 2004; Institute of Medicine 2005; Leyden 2003). In addition, neighborhood problems (e.g., traffic, noise, crime, inadequate lighting, and lack of public transportation) have been associated with increased risk for loss of physical functioning (Balfour and Kaplan 2002; Krause 1998). In summary, less-walkable neighborhoods appear to be linked to reduced physical functioning in community-dwelling elders.

## Built environment, social support, and health

Besides the direct impact of built environment on physical functioning, one possible mechanism by which neighborhoods may affect residents’ behavior and health is creating or limiting opportunities for social support from the neighborhood (Thompson and Krause 1998). Social support has been found to be an important determinant of elders’ health and mental health (House et al. 1988; Krause et al. 1989), and direct, face-to-face contact is associated with greater perceived availability and adequacy of support (Thompson and Krause 1998). Consequently, if physical characteristics of neighborhoods inhibit contact, obtaining support may be more challenging, particularly in older residents (Thompson and Krause 1998).

Several studies have suggested that the built environment can facilitate or impede social support. For example, older adults living in deteriorated neighborhoods reported lower social support than did those living in well-maintained neighborhoods, which may partly account for the reduced health of elders in deteriorated neighborhoods (Krause 1996). Other work suggests that certain built-environment characteristics—such as walkable, mixed-use neighborhoods—are associated with increased social capital, or social networks and interactions that inspire reciprocity and trust (Leyden 2003). In turn, social capital and the related construct of collective efficacy (i.e., social cohesion plus informal social control) predict several positive health outcomes, including reduced levels of mortality, depression, and violence (Kawachi 1999; Sampson et al. 1997). Evidence on the interconnections of the built environment, social support, and health is therefore accumulating, but little work has examined these relationships simultaneously in any one population (Srinivasan et al. 2003).

## The present investigation

Relevant to the above research, Jane Jacobs (1961) and other theorists of urban life (Leccese and McCormick 2000) have proposed that positioning buildings with windows, porches, and stoops close to the street or other public space promotes a bond among neighbors, who share a sense of ownership of that space. Additionally, it has been proposed that these features may promote increased social interaction and heightened social support (Evans 2003; Jacobs 1961). Increased social support in turn may lead to enhanced mental health and possibly physical health outcomes (Evans 2003; Jacobs 1961; Spokane et al. 2007). For example, satisfaction with social support has been found to predict depressive symptoms over time in elders (Krause et al. 1998). Improvements in mental health may themselves lead to better physical functioning over time (Penninx et al. 1998). Hence, built-environment features theorized to encourage observations and interactions may promote elders’ physical functioning either directly, by encouraging mobility (e.g., climbing steps on a stoop), or indirectly, through their effects on social support and psychological health.

Given this literature, in a population-based sample of lower-socioeconomic-status (SES) elders, we examined *a*) whether built-environment features theorized to promote observation and interaction are associated with elders’ subsequent physical functioning and *b*) whether any observed relationships between the built environment and physical functioning occur, at least in part, through the relationships between the built environment and social support and psychological distress (Spokane et al. 2007). To our knowledge, this is the first study to evaluate all of these sequential relationships in a single model.

## Materials and Methods

### Research design

We conducted this study as part of a larger, prospective cohort study on the relationship between the built and social environment and Hispanic elders’ mental and physical health outcomes. This study was approved by the University of Miami’s Institutional Review Board (IRB). We complied with all applicable federal and state guidelines (including IRB requirements), and all participants gave written informed consent before the study. We conducted the study in East Little Havana, a low-SES, predominantly Hispanic urban community in Miami, Florida. East Little Havana is 93% Hispanic, with 19% of residents ≥65 years of age, and 35% of residents living below the poverty level (U.S. Census Bureau 2000). This neighborhood includes 3,857 lots in 403 blocks, with 40,865 residents in 8 km^2^ (U.S. Census Bureau 2000). We selected East Little Havana because its sociodemographics are relatively homogeneous [i.e., mostly Hispanic, with the highest rate of poverty in the county (U.S. Census Bureau 2000)], yet its built environment demonstrates considerable variability. For instance, built-environment features, such as porches and windows, that have been theorized to facilitate observation and social interaction (Jacobs 1961; Leccese and McCormick 2000) were differentially present across the blocks of this neighborhood, ranging from blocks without any porches or windows facing the street to blocks with many porches and windows facing the street (Spokane et al. 2007).

In 2000–2002, we assessed each lot in East Little Havana for built-environment features. We then conducted a door-to-door survey enumerating all 16,000 households, identifying 3,322 community-dwelling Hispanic elders ≥70 years of age. Elders meeting these criteria lived on 302 of the 403 blocks comprising East Little Havana.

We randomly selected one Hispanic elder from each block on which elders lived. If an elder refused to participate or did not meet inclusion criteria, we approached a second randomly selected elder, and so on, until one elder in each of the blocks with elders agreed to participate. Through this process, we ultimately considered 521 elders for possible participation. But of this total, 30 died, 95 refused, 80 moved away, 10 had incorrect home addresses, 24 did not meet other eligibility criteria [the primary reason for exclusion was low scores on the Mini-Mental State Examination (Folstein et al. 1975)], 7 could not be contacted after 11 home visits, and 2 moved to blocks from which we had already sampled elders. The final sample at baseline consisted of 273 eligible elder participants living one each in 273 blocks (i.e., we were unable to sample elders from 29 of the 302 blocks on which elders resided, despite random resamplings from the same block, which occurred primarily because most of these 29 blocks had few elders residing on them).

As part of the larger study, the participants completed three annual assessments of social support and mental health (i.e., psychological distress, cognition), with most baseline assessments completed in 2002–2003. Beginning with the 24-month assessments in 2004–2005, the participants also completed measures of physical functioning.

### Participants

Inclusion/exclusion criteria were as follows: *a*) ≥70 years of age, *b*) born in a Spanish-speaking country, *c*) resident of East Little Havana, *d*) living in housing from which s/he can walk outside (this would exclude nursing homes or specialized locked housing units), *e*) of sufficient physical health to go outside without physical assistance from another person, based on reports from the participant and a trained assessor, and *f*) scored ≥17 on the Mini-Mental State Examination (Folstein et al. 1975). We paid participants $25, $30, and $35, for the baseline, 12-month, and 24-month interviews, respectively.

### Measures

**Figure 1:**
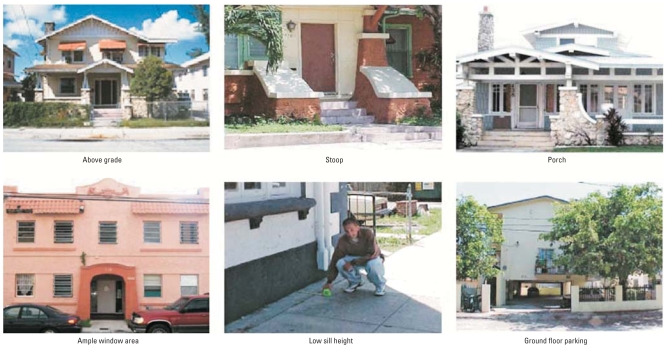
Illustrations of selected built-environment variables. See “Materials and Methods” for explanation of terms.

We measured the “built environment” of the study area using the University of Miami Built Environment Coding System (UMBECS), a comprehensive coding system developed to assess urban constructs (Leccese and McCormick 2000; Lombard et al. 2000; Spokane et al. 2007). The UMBECS coding system is supported by a manual (Lombard et al. 2000) that operationalizes specific features (e.g., commercial frontage, porches and stoops, sidewalk width) related to urbanist constructs (e.g., diversity of use, visibility from interior and exterior, street/sidewalk walkability). To increase reliability, each code is operationalized and illustrated with a photograph (Figure 1). We trained architecture students to an inter-rater reliability of 0.80. The final UMBECS measure and manual consisted of 76 built-environment features (Lombard et al. 2000; Spokane et al. 2007). We then coded each of the 3,857 lots in all 403 blocks of East Little Havana, which included households as well as all other land uses in the community (e.g., commercial uses), using the UMBECS. For purposes of the present study, we coded each lot on the following seven indicators assessing “eyes on the street” (Figure 1):

- Above grade: buildings that “sat” at least 0.30 m above the level of the sidewalk
- Stoop: a small raised platform at the entrance of a building, typically composed of several steps, which provides a place for sitting
- Porch: any covered exterior space protecting the entrance to a building
- Window area: the proportion of building face composed of windows
- Low-sill-height windows (< 0.91 m from the sill of the dominant first floor window to the main level of the first floor), which allow occupants to more easily see out to the street
- Ground-floor parking: the ground floor of a multistory building dedicated to parking (hypothesized to be detrimental)
- Setback: the distance from the building to the street (smaller setback being preferred; for analytic purposes, we reverse-scored this variable and term it “small setback”).

We weighted data regarding these seven features according to the frontage of the lot (i.e., the proportion of the block face corresponding to each lot) and aggregated data for each block in East Little Havana. The resulting variable was an estimate of the proportion of the total block frontage for which each built-environment feature was present. [For further details regarding the UMBECS coding system, see Spokane et al. (2007) and the UMBECS manual (Lombard et al. 2000)].

We assessed social support using the Spanish-language translation of three scales (Krause 1995; Krause and Markides 1990), which are highly correlated with each other and with elders’ mental health (Krause 1995; Krause et al. 1989): *a*) satisfaction with support over the last month consisted of three, four-choice Likert items (e.g., “In general, how satisfied are you with the help you have received in the last month with transportation, household chores, gardening, and shopping?”); *b*) satisfaction with support over the last year consisted of three, three-choice Likert items (e.g., “During the past year, do you feel that this type of help was provided often enough, or do you wish it was given to you more often or less often?”); *c*) negative interactions consisted of four, four-choice Likert items tapping negative social interactions during the past month (e.g., “In the past month, how often have others pried into your affairs?”). Reliability estimates for the Spanish translation of these scales were acceptable for this study population, with α-values of 0.71, 0.73, and 0.73, respectively. (For further information about the social support scales, please contact the authors.)

We measured psychological distress by self-reported anxiety and depressive symptoms in the past week, and measured anxiety using a 10-item, Spanish version of the Spielberger State Trait Anxiety Inventory (Spielberger et al. 1983), which has adequate reliability and validity (for the Spanish translation of this scale with this sample, α = 0.89) (Novy et al. 1995). We assessed depressive symptoms using the seven-item Depressive Affect sub-scale of the Center for Epidemiological Studies Depression Scale (Radloff 1977), which reliably assesses elders’ underlying mood symptomatology while excluding somatic symptoms (e.g., difficulties sleeping) that may be partly confounded with age and physical health (for the Spanish translation of this subscale with this sample, α = 0.79) (Fonda and Herzog 2001).

We assessed physical functioning by three measures shown to be interrelated in previous studies (Brach and VanSwearingen 2002; Femia et al. 2001; Jylhä et al. 2001): *a*) grip strength, or the amount of force the elder exerts with each hand using a hand dynamometer (Jylhä et al. 2001) (average of two measurements per hand; α = 0.98 for this sample); *b*) gait speed, or the elder’s speed (meters per second) in walking a 12-foot walking course at one’s usual pace (Brach and VanSwearingen 2002; Jylhä et al. 2001) (average of two measurements; α = 0.99 for this sample); and *c*) self-reported health, assessed by two questions (i.e., health “in general” and “at present”) on a five-point Likert scale each (i.e., 1 = poor; 5 = excellent), that are highly correlated (*r* = 0.83 for the Spanish translation of these items used in this sample) and are related to objective health and functioning [e.g., self-rated health is positively correlated with grip strength and gait speed (Femia et al. 2001; Jylhä et al. 2001)]. In addition, self-rated health has been conceptualized as a summary expression of the various functional impairments known to the individual respondent (Tissue 1972), and functional impairments have been shown to be a significant component of self-rated health in much prior work (Han et al. 1999; Liang 1986).

### Analytic strategy

**Figure 2:**
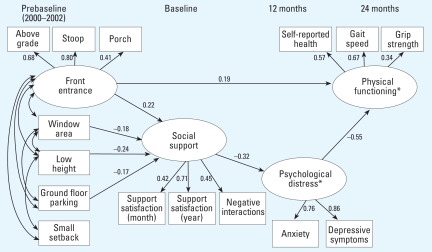
Structural equation model of the relationship of built-environment “eyes on the street” features to social support, psychological distress, and physical functioning: standardized β estimates are reported for each path, significant at *p* < 0.05, and standard fit indices are reported for the overall model, which suggest acceptable model fit (Arbuckle 2005; Kline 2005). χ^2^ (df 121) = 161.28, *p* < 0.001; χ^2^/df = 1.333; comparative fit index (CFI) = 0.95; root mean square error of approximation (RMSEA) = 0.035. *Controls for sex, age, and income.

The present analyses examined the sequential relationships among each of the following: *a*) built-environment features measured at prebaseline (i.e., 2000–2002), *b*) social support measured at baseline (i.e., beginning in 2002), *c*) psychological distress measured at 12 months, and *d*) physical functioning measured at 24 months. Thus, the four main variables in the analyses (i.e., built environment, social support, psychological distress, and physical functioning) corresponded to each of four different time points. We selected this temporal ordering *a*) to examine the hypothesized direct impacts of the built environment at prebaseline on subsequent physical functioning at 24 months (the first time point at which physical functioning data were available), and *b*) to examine the hypothesized indirect impacts of built environment on physical functioning, through its sequential impacts on social support at baseline and psychological distress at 12 months, respectively (Figure 2).

We tested the hypothesized relationships using structural equation modeling, which involves positing a model based on specific, *a priori* defined relationships between variables that create patterns that should be observed in a covariance matrix. We then assessed and evaluated the degree of correspondence between observed and predicted covariance matrices. Structural equation modeling has several strengths, including the ability to directly test complex mediational relationships and to address data characteristics (e.g., correlated errors) that would violate assumptions for other techniques, such as multiple regression or analysis of variance.

We conducted structural equation modeling of the relationship between the built environment at prebaseline and social support at baseline, psychological distress at 12 months, and physical functioning at 24 months, using AMOS 6.0 statistical software (Arbuckle 2005). These structural equation modeling analyses controlled for the relationship between age, sex, and income and psychological distress and physical functioning, because these sociodemographic variables have been shown to be important correlates of psychological distress and physical functioning in elders (e.g., Femia et al. 2001; Narrow et al. 1990; Ottenbacher et al. 2005; Rantanen et al. 1999). We addressed missing data using the full information maximum likelihood algorithm (Arbuckle 2005). Finally, to provide additional depth and practical interpretability to the findings, we conducted a supplementary analysis using binomial regression to predict the risk of poor physical functioning based on the built-environment features (Spiegelman and Hertzmark 2005).

## Results

### Preliminary analyses

Preliminary analyses first examined the participants’ characteristics on both sociodemographics and the main analytic variables of interest (Table 1). At baseline, the final sample was 59% female and 87% Cuban born, with a mean age of 78.5 years and a mean (± SD) annual household income of $9,300 ± $4,550. At baseline, participants reported living in their home for an average of 13.7 ± 11.3 years; 34% were married, and 7% were employed. By the 12-month follow-up interview, we lost 39 of the original 273 participants to follow-up (19 died, 9 refused, 5 moved out of greater Miami, and 6 could not be located). By the 24-month interview, we lost an additional 17 participants to follow-up (six died, two refused, two moved out of greater Miami, and seven could not be located). Those whom we lost to follow-up were older, more likely to be male, and lower in support satisfaction at baseline, compared with those whom we recontacted at 24 months. There were no other significant differences between these groups on the predictor variables of interest, and further analyses (see “Limitations” under “Discussion”) suggested that loss of participants to follow-up did not significantly affect the main study findings.

Preliminary analyses next examined whether constructs could be represented by one or more latent variables, which are unmeasured factors or constructs that are estimated via two or more observed variables. In essence, they can be viewed as a weighted index based on multiple indicators where the weights correspond to the degree to which individual indicators reflect the underlying common construct. Psychometrically, latent variables provide more reliable estimates of underlying constructs of interest and allow researchers to separate measurement error from estimates of the construct itself (Kline 2005; Loehlin 2004).

Table 2 shows the zero-order correlations among the built-environment variables. Based on the corresponding covariances, we developed a model in which these features were reflected in one latent variable and four manifest (observed) variables. The latent variable “front entrance” refers to the relationship of the building’s entrance to the street and consists of the proportion of a block in which buildings were above grade, had a stoop, and had a porch. The other four built-environment variables (window area, low sill height, ground-floor parking, and small setback) had weaker and less consistent relationships with each other and with the three front entrance variables, so we considered them separately, as individual variables in the model (Table 2).

In contrast, we had an *a priori* plan to create specific latent variables for social support, psychological distress, and physical functioning based on the multiple indicators of each, assuming that the corresponding indicator variables were sufficiently correlated. The last-month and last-year measures of support satisfaction were significantly correlated at baseline (*r* = 0.31, *p* < 0.001) and were each correlated with the reverse-scored measure of negative interactions (1 year: *r* = 0.30, *p* < 0.001; 1 month: *r* = 0.16, *p* < 0.01). In the interest of a more parsimonious model, we combined these three measures into a single latent variable, “social support.” The measures of anxiety and depressive symptoms were significantly correlated at the 12-month interview (*r* = 0.66, *p* < 0.001), and we combined them into the latent variable “psychological distress.” Finally, grip strength and gait speed were significantly correlated at 24 months (*r* = 0.39, *p* < 0.001) and were each correlated with self-reported health (grip strength: *r* = 0.30, *p* < 0.001; gait speed: *r* = 0.41, *p* < 0.001). Consequently, we incorporated these three measures into the latent variable “physical functioning.”

Because latent variables are an amalgamation of multiple measured variables, they have no inherent metric (Kline 2005; Loehlin 2004). Furthermore, because we created the latent variable scores based on multiple indicators, blocks may have the same front entrance score because of different combinations of the corresponding built-environment features. For example, assuming all else is equal, a block would receive the mean front entrance score if 30% of the frontage had a stoop, 43% was above grade, and 9% had a porch. Alternatively, the same score would be obtained even if a block had no stoops but a higher portion of the frontage that was above grade and/or had a porch (88% and 29%, accordingly). In effect, different blocks could obtain the same latent variable score through different combinations of high, medium, or low values on the corresponding manifest variables. Together, these issues can make the interpretation of effects involving latent variables difficult. Therefore, Table 3 provides examples of different ways that blocks could receive front entrance and physical functioning latent variable scores at the mean and 1 SD above and below the mean. This can help to provide context regarding what differences in these scores reflect in the “real world.”

Using these latent variables and based on the literature (Abu-Ghazzeh 1999; Balfour and Kaplan 2002; Clarke and George 2005; House et al. 1988; Jacobs 1961; Krause et al. 1989; Leccese and McCormick 2000; Leyden 2003; Penninx et al. 1998; Spokane et al. 2007; Szapocznik et al. 2006), we developed a preliminary model in which we predicted the built-environment features to have a direct relationship with physical functioning at 24 months, and an indirect relationship with physical functioning through social support and psychological distress. In other words, we theorized prebaseline built-environment features to predict both *a*) physical functioning at 24 months and *b*) social support at baseline. We theorized social support at baseline to predict psychological distress at 12 months, which in turn would predict physical functioning at 24 months. We obtained social support, psychological distress, and physical functioning measures at baseline, 12-, and 24-month study assessments, respectively.

In this model, we allowed the front entrance latent variable and the remaining four built-environment manifest variables to covary, and included age, sex, and income as control variables for psychological distress and physical functioning. Finally, because sex had a substantially stronger correlation with grip strength (*r* = 0.73, *p* < 0.001) than with the other physical functioning indicators (*r*-values < 0.25), we also included it as a control variable for grip strength.

### Final model

On the basis of the preliminary model, we conducted subsequent analyses that sequentially eliminated all nonsignificant pathways and covariances to derive a final model (Figure 2). Eliminating the nonsignificant pathways had no meaningful impact on the statistical significance or stability of the relationships presented in Figure 2. The difference between the preliminary model and the final model was not statistically significant [χ^2^ (7 df) = 7.52, *p* > 0.37]. We therefore selected the more parsimonious model with the fewest number of pathways, as depicted in Figure 2 (all paths presented were statistically significant, *p* < 0.05). The computed fit indices (Figure 2) indicated an acceptable fit of the overall model to the data (Arbuckle 2005; Kline 2005). [We used standard fit indices for structural equation modeling to evaluate the fit of the overall model to the data: values of χ^2^/df< 3, comparative fit index (CFI) > 0.90, and root mean square error of approximation (RMSEA) < 0.08 suggest adequate model fit (Arbuckle 2005; Kline 2005).]

In the final model, the front entrance latent variable had a direct and positive relationship with physical functioning, indicating that elders who lived on blocks with greater proportions of frontage that included porches, stoops, and buildings built above grade had higher performance on the physical functioning measures. However, none of the four manifest built-environment variables (window area, low sill height, ground-floor parking, and small setback) were directly related to physical functioning.

In contrast, almost all of the built-environment variables were related to social support. The front entrance latent variable was predictive of higher levels of social support, indicating that elders who lived on blocks with greater proportions of porches, stoops, and buildings built above grade reported higher scores on the social support variables. In addition, three of the four manifest built-environment variables were also associated with social support, albeit in a negative direction. As expected, ground-floor parking was negatively related to social support, with elders living on blocks with less ground-floor parking reporting higher levels of social support. Unexpectedly, window area and low-sill-height were also negatively related to social support. The remaining built-environment feature, small setback, was not significantly related to social support.

Regarding pathways further “downstream” in the model, as expected, social support was strongly related to psychological distress, with those elders reporting higher levels of social support reporting lower levels of depressive symptoms and anxiety. Also as expected, psychological distress in turn was strongly associated with physical functioning.

Although the overall model reflected the complex interrelationship of the built environment, the social environment, psychological distress, and physical functioning (Figure 2), we conducted a final series of supplementary analyses to gain additional depth and practical interpretability for the effect of a negative built environment as a predictor of poor physical functioning. For these supplemental analyses, we performed a pair of binomial regressions predicting poor physical functioning based on the built-environment front entrance features. In order to perform these analyses, we calculated regressed factor scores for both the physical functioning and front entrance latent variables. We then dichotomized each of these scores at each of the corresponding 10th percentiles (≤10th percentile coded as 1; > 10th percentile coded as 0). The first binomial regression included only the dichotomized front entrance variable as a predictor. This analysis suggested that elders living on blocks marked by low levels of positive front entrance features were 2.7 times as likely to have poor subsequent physical functioning compared with elders living on blocks with greater numbers of positive front entrance features [*b* = 0.99; χ^2^ (1 df) = 3.71; *p* = 0.05; 95% confidence interval (CI), 1.0–7.3]. The second binomial regression included the dichotomized front entrance variable but also controlled for levels of anxiety and depression, which had served as partial mediators in the structural equation model; this resulted in a statistically significant effect for front entrance [*b* = 1.31; χ^2^ (1 df) = 5.54; *p* = 0.02; 95% CI, 1.3–11.1], indicating that controlling for anxiety and depression, elders living on blocks marked by low levels of positive front entrance features were 3.7 times as likely to have subsequent poor physical functioning, compared with elders living on blocks with greater numbers of positive front entrance features.

## Discussion

The results of this study suggest that architectural features of the built environment that are believed to promote visual and social contacts among residents—termed “eyes on the street” (Jacobs 1961)—were significantly associated with elders’ subsequent physical functioning. As predicted, elders who resided on blocks with more front porches, stoops, and buildings built above grade had significantly better physical functioning at 24-month follow-up than did elders who resided on blocks with fewer of these architectural features. Although these same three “front entrance” built-environment features were associated with higher levels of social support at baseline, which sequentially were associated with psychological distress and physical functioning, almost all of the total relationship between “front entrance” features and physical functioning was attributable to their direct relationship with physical functioning. In contrast, three other built-environment features—window area, low sill height, and ground-floor parking—had only indirect and negative relationships with elders’ physical functioning at 24 months.

Additional analyses revealed that almost all (84%) of the total relationship between the “front entrance” variable and physical functioning was attributable to its direct relationship with physical functioning, with the indirect pathway through social support and psychological distress accounting for the remaining 16%. In contrast, three of the four manifest built-environment variables were related to physical functioning only indirectly, through their associations with social support and psychological distress, which in turn predicted physical functioning. Further analyses showed that the presence of the built-environment variables in the final model accounted for an additional 8% variance in physical functioning compared with a model in which the only predictors of physical functioning were social support, psychological distress, and the demographic/control variables, which at a population level could represent a very meaningful impact.

The direct relationship between “front entrance” features (i.e., porches, stoops, and buildings built above grade) and physical functioning may reflect several possible processes. Such features may encourage mobility to the feature itself (e.g., climbing stairs to sit on a stoop) or to the adjoining outdoor environment (e.g., walking across the street to chat with a neighbor seen from one’s front porch) (Balfour and Kaplan 2002). Alternatively, neighborhoods may be more “walkable” when they permit more face-to-face interactions and monitoring for safety (Jacobs 1961; Leccese and McCormick 2000), and hence enhance physical functioning by encouraging physical activity (Balfour and Kaplan 2002; Institute of Medicine 2005). Both interpretations are consistent with the finding that residents walk more in neighborhoods with pre-1945 construction (which include more porches and stoops) than in neighborhoods with more recent construction (which includes ground-floor parking) (Berrigan and Troiano 2002; Leccese and McCormick 2000). In fact, follow-up analyses revealed that, in our study area of East Little Havana, buildings with more positive front-entrance features (e.g., porches) tended to be older (pre-1945) than buildings with fewer of these features (e.g., absence of porches).

To enhance the interpretability of some of the specific relationships described in the structural equation model (e.g., a 1-SD increase in the front entrance latent variable is associated with a 0.19-SD increase in physical functioning), it may help to consider how the built environment and physical functioning latent variables translate into real-world characteristics of individual blocks and elders. (We based the following examples on actual blocks and elders, but the specific values have been modified slightly to protect participant confidentiality.) Specifically, an example of a block at the mean of the front entrance latent variable is one where 36% of the frontage has buildings that are above grade and 36% has stoops. In contrast, an example of a block that is 1 SD below the mean has only 15% of its frontage above grade and only 10% with stoops, whereas a block that is 1 SD above the mean has 67% of its frontage above grade and 50% with stoops. Similarly, a hypothetical elder who is at the mean for the physical functioning latent variable has a gait speed of 0.59 m/sec and grip strength is 40.75 kg, whereas an elder who has a value 1 SD below the mean has a gait speed of 0.48 m/sec and grip strength of 18.75 kg, and an elder who is 1 SD above the mean has a gait speed of 0.72 m/sec and grip strength is 64.5 kg. These illustrations suggest that relatively modest differences in built-environment features may be related to modest but significant variations in elders’ physical functioning.

In contrast, the other built-environment features examined in this study (ground-floor parking, window area, and low sill height) may limit social interactions that are necessary for positive health outcomes by failing to achieve the proper balance between public and private space. On the one hand, buildings with ground-floor parking may severely limit residents’ visual and social access to pedestrians on the street; on the other hand, low sill height may be “too much of a good thing” by allowing neighbors to see into one’s own home and may cause the house dweller to engage in behavior (e.g., shutting the blinds) that precludes further social interaction. This result is reminiscent of prior work suggesting that sufficient balance needs to be maintained between residents’ needs for privacy and public spaces for maintaining optimal psychosocial functioning (e.g., Kweon et al. 1998; Skjaeveland and Garling 1997; Weich et al. 2002).

Even more important, to enhance the interpretability of the findings, we conducted supplemental analyses to further assess the potential effect of a negative built environment on poor physical functioning in elders. These analyses, which used binomial regression, suggested that elders living on blocks with few positive front entrance variables were 2.7 times as likely to have subsequent poor levels of physical functioning compared with elders living on blocks with a greater number of positive front entrance features. The findings of this and other studies suggesting that the neighborhood built environment may be an important determinant of health in older persons (Balfour and Kaplan 2002; Clarke and George 2005; Krause 1998) point to the need for more research to identify the specific environmental characteristics that may best promote elders’ physical functioning and independence.

### Limitations

This study has several limitations. First, the inherent inability to randomly assign elders to blocks allows for potential self-selection bias. For example, although we statistically controlled for SES, higher-income elders may have chosen to live in more desirable blocks with “better” built environments, which in turn could account for the relationship between built-environment characteristics and physical functioning. However, this self-selection bias would work against our hypotheses, given that several of the “eyes on the street” features (e.g., porches, stoops) tend to occur in homes built before 1945, whereas more desirable buildings may tend to be newer and have less salutary built-environment characteristics (e.g., absence of porches, presence of ground-floor parking). Similarly, the elders who were either the most physically active or the most sociable may have chosen to move to homes with front entrances that supported their physical functioning or social interaction. Future research is therefore needed to disentangle the possible influence of self-selection from the influence of the built environment on elders’ physical functioning over time. Additionally, future studies should consider other variables (e.g., neighborhood characteristics such as crime and pedestrian safety, and individual characteristics such as social skill) that may mediate or moderate the relationship between built-environment characteristics and residents’ health.

In addition, social support, psychological distress, and health status were self-reported, and unmeasured personality characteristics or attributional styles may have affected those measures. Similarly, although the physical assessments included gait speed and grip strength, which are frequently used measures of physical functioning in the literature (Femia et al. 2001; Jylhä et al. 2001), we did not measure other factors that may be related to physical functioning, such as flexibility, aerobic capacity, or pain in movement, because of the inherent limitations of conducting a 3-hr in-home assessment in a population of very old adults. Moreover, we based this work on elders living in a single community, requiring replication in both similar and fundamentally different communities. Similarly, the present research examined effects of the built environment at the block level, and future research should consider the health effects of the built environment at other geographic levels of analysis (e.g., the home level, or neighborhood level). Furthermore, although we obtained longitudinal data, these covered only a 3-year period, and physical functioning was available only at the final time point. This precluded us from examining the impacts of built and social environments on changes in health over time. Nevertheless, the available data enabled us to examine potential sequential relationships from built and social environments to elders’ physical functioning as measured ≥2 years later.

A further limitation is that > 40% of older adults randomly selected for participation were not enrolled, primarily due to deaths, refusals, and moves away from the study area. Finally, attrition occurred over time, which had the potential to affect the study results. For example, those lost to follow-up were older, more likely to be male, and lower in support satisfaction at baseline compared with those recontacted at 24 months. However, we reconducted the main analyses removing the 25 participants who died before 24 months and obtained similar results for the final structural equation model (Figure 2), with all fit indices in the acceptable range [χ^2^ (df 121) = 162.30, χ^2^/df = 1.34, CFI = 0.95, RMSEA = 0.037], and all paths statistically significant except for the path from window area to social support (*p* = 0.099). We obtained similar results for the final model (Figure 2) after removing all 56 participants whom we lost to follow-up at 24 months.

### Strengths

Several strengths inherent in this study should be noted. This is one of the first studies to explore both neighborhood physical conditions (i.e., built environment) and social conditions (i.e., social support) as possible protective factors for physical functioning, and we did so in a population-based sample of low-SES Hispanic elders, who are at greater risk for disability than other racial/ethnic groups (Schoeni et al. 2005). In addition, this is the first study of which we are aware to show that block-level built-environment variables predict health. Moreover, this study captured fine-grained lot-level information on the built environment in the area immediately around the individual’s home, which may be especially applicable to elders rather than arbitrary Census-defined boundaries (Berke et al. 2007). Furthermore, the study uses objective measures of built environment [i.e., UMBECS (Lombard et al. 2000; Spokane et al. 2007)] and physical functioning (i.e., grip strength, gait speed). The temporal nature of the data collection provides an additional strength: we collected built-environment data before the psychosocial and physical assessments, allowing for greater confidence in judgments of causality regarding the effects of built environment on psychosocial and physical functioning. Finally, despite the rather restricted nature of this sample, we identified substantial variability in built environment and social support, both of which predicted psychological distress and physical functioning.

## Conclusions

In summary, findings suggest that architectural features believed to promote observation and interaction (Jacobs 1961; Leccese and McCormick 2000) may have an important impact on elders’ physical functioning. To our knowledge, this is the first analysis to show a relationship between block-level built-environment features and residents’ health. Although preliminary, these results add to the existing literature suggesting that the built environment may be a factor in elders’ physical functioning (Balfour and Kaplan 2002; Clarke and George 2005; Krause 1998). Future research should identify interventions (i.e., neighborhood redesign) by which elders could maintain a high level of functioning and “age in place” for as long as possible without need for institutionalization.

## Figures and Tables

**Table 1:** Distributional statistics of measured indicators in the final model shown in Figure 2.

	Observation	
Variable	Mean ± SD	Range
Built-environment variables^a^		
Front entrance variables		
Above grade	0.44 ± 0.25	0–1
Stoop	0.30 ± 0.21	0–0.91
Porch	0.09 ± 0.11	0–0.55
Window area	0.63 ± 0.17	0.10–0.98
Low sill height	0.44 ± 0.23	0–1
Ground-floor parking	0.02 ± 0.06	0–0.50
Small setback^b^	0.25 ± 0.18	0–0.99
Biopsychosocial variables		
Social support (baseline)		
Support satisfaction (month)	7.61 ± 2.13	0–9
Support satisfaction (year)	5.66 ± 0.89	3–9
Negative interactions^c^	9.89 ± 2.05	0–11
Psychological distress (12-month)		
Anxiety	19.74 ± 7.61	10–40
Depressive symptoms	4.36 ± 4.27	0–18
Physical functioning (24-month)		
Self-reported health^d^	2.62 ± 0.83	1–5
Gait speed (m/sec)	0.60 ± 0.21	0.07–1.09
Grip strength (kg)	36.61 ± 16.53	7.75–96.5
Demographic covariates		
Age (years)	78.48 ± 6.32	70–100
Female sex (%)	59	
Income (US$)	$9,300 ± 4,550	$2,500–55,000

a Values indicate the proportions of block frontage that correspond to each built-environment feature.

b Based on the observed distribution, we capped setback at 6.1 m and rescaled it proportionally from 0 to 1 within this range. We reverse scored this variable and termed it “small setback,” so that higher values correspond to shorter distances from the building to the street.

c We reverse scored negative interactions so that higher values correspond to fewer negative interactions.

d We rated self-reported health on a scale from 1 (poor) to 5 (excellent).

**Table 2:** Zero-order correlations among built-environment variables.

	Grade	Stoop	Porch	Windows	Sill	Ground parking	Small setback
Grade	—						
Stoop	0.54**	—					
Porch	0.26**	0.35**	—				
Windows	0.36**	0.35**	0.11	—			
Sill	0.16**	0.25**	0.13*	0.38**	—		
Ground parking	−0.11	−0.20**	−0.08	−0.20**	−0.09	—	
Small setback	−0.17**	−0.15*	−0.12*	−0.03	0.22**	−0.02	—

* *p* < 0.05.

** *p* < 0.01.

**Table 3:** Illustrative examples of front entrance features and physical functioning data that would produce various latent variable factor scores.

	Front entrance^a^			Physical functioning		
Measure	Porches	Stoops	Above grade	Self-report health^b^	Gait speed (m/sec)	Grip strength (kg)
Example 1						
−1 SD	0.01	0.10	0.19	1.88	0.41	21.89
Mean	0.09	0.30	0.43	2.66	0.61	37.27
+1 SD	0.19	0.50	0.66	3.43	0.81	52.73
Example 2						
−1 SD	0.05	0.00	0.35	2.19	0.49	8.00
Mean	0.29	0.00	0.88	3.30	0.77	8.00
+1 SD	0.98	0.00	1.00	4.42	1.06	8.00

a Values indicate the proportions of block frontage that correspond to each built-environment feature.

b We rated self-reported health on a scale from 1 (poor) to 5 (excellent).
